# Hospital design for better infection control

**DOI:** 10.4103/0974-2700.55329

**Published:** 2009

**Authors:** Fatimah Lateef

**Affiliations:** Department of Emergency Medicine, Singapore General Hospital, Outram Road, Singapore

**Keywords:** Emerging infection, infection control measures

## Abstract

The physical design and infrastructure of a hospital or institution is an essential component of its infection control measure. Thus is must be a prerequisite to take these into consideration from the initial conception and planning stages of the building. The balance between designing a hospital to be an open, accessible and public place and the control to reduce the spread of infections diseases is a necessity. At Singapore General Hospital, many lessons were learnt during the SARS outbreak pertaining to this. During and subsequent to the SARS outbreak, many changes evolved in the hospital to enable us to handle and face any emerging infectious situation with calm, confidence and the knowledge that staff and patients will be in good stead.

This paper will share some of our experiences as well as challenges

## INTRODUCTION

When the statutes of the hospital of St John Bridgewater were developed in 1219, Bishop Joscelin of Bath commented -, “No lepers, lunatics or persons having the following sickness or other contagious diseases are to be admitted to the house, and if any such be admitted by mistake, they are to be expelled as soon as possible”.[1] Hospitals and healthcare institutions have certainly come a long way from the days of Bishop Joscelin. We are not as drastic in our sentiments today and we do not expel patients with infectious diseases. In fact, we admit them to suitably planned facilities and rooms and ensure that they do not cause unnecessary hazards to staff and other hospital users.

The physical design of a hospital is an essential component of its infection control measures to minimize the risk of transmission of any infectious disease. When historical and traditional hospitals were built, there were minimal concerns of new emerging infectious diseases. Today, with a more progressive outlook, it is the fundamental requirement to adopt a holistic view of the design and management of hospitals. Designing hospitals to be open, public spaces can make it difficult to control the spread of infectious diseases. The ease of travel and transportation today helps people cross borders easily. They can harbor, carry or catch infectious agents readily. During the Severe Acute Respiratory Syndrome (SARS) outbreak it became clear that the multiple public entrances in hospitals make it difficult, and often costly, to control entry and thus infiltration of infectious diseases.[2,3]

Only a few hospitals have an adequate supply of isolation and negative pressure rooms in wards, emergency departments (EDs) and Intensive Care Units (ICUs). While hospitals may not have complete control over host factors and agents, they are still responsible for the environment that surrounds the patients. By controlling and ensuring adequate sanitization of the environment of the host, hospital authorities can reduce the incidence of hospital acquired infections.

A decision on hospital buildings must be based on multiple factors besides cost, like fire protection, strength of construction material, hygiene, building health, environmental protection, sound isolation, energy saving, durability and utilization rate, among others. Even after initial completion of the hospital building, systematic data collection and feedback for addition, modification and upgrading of the infrastructure must be ongoing.[4] Built-in flexibility in design is becoming more crucial, mainly because technology is quickly obsolete and patient population is constantly changing. For example, single rooms may be more useful to have as they can be converted to isolation rooms more readily during an outbreak. Healthcare buildings are a complex environment with a need for specialized areas like high wear and tear areas, circulation areas, wards, specialized theatres and hazardous material chain of disposition. Choice of material and finish is also important and needs to be mainstreamed into the planning stages.[2,4]

With the challenges of new and emerging infectious diseases as well as higher public expectations and awareness of healthcare related issues, much consideration has to be given to these in the planning phase of building hospitals. For existing institutions and hospital buildings, renovation and upgrading plans must incorporate the necessary changes. Among the various methods for infection control two important environment factors are isolation and ventilation. Infected patients or those highly susceptible to infection need to be isolated in private rooms with proper ventilation systems in order to stop spread and reduce the possibility of developing a new infection. Bronson Methodist Hospital in Michigan demonstrated that private rooms, location of sinks and air flow design have resulted in a 10-11% decline in overall nosocomial infections rate.[5]

## THE SINGAPORE GENERAL HOSPITAL WAY

Following the experience of SARS in 2003, infectious diseases and potential infectious patients are now being managed with high vigilance in an upgraded infrastructure at Singapore General Hospital. At points of entry into the hospital and in the ED, patients are screened using a rapid questionnaire on their travel exposure, fever history and symptoms. Body temperature is recorded and documented. Any one with fever and a positive response to any question will be channeled to be managed in the febrile area of the ED. This febrile screening step is done outside the ED in a specially planned area before formal ED triage is done. The rationale is to identify the high risk patients as soon and as early as possible. Other points of entry into the hospital are also regulated, especially during high risk periods.

### Patient management in fever area

**Figure 1:**
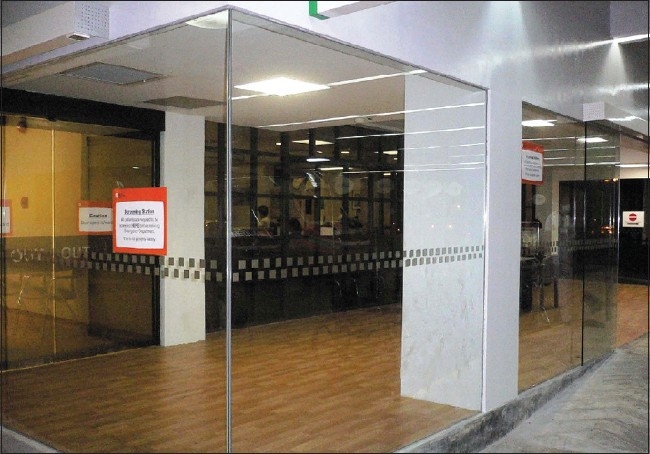
The separate screening area for febrile patients

**Figure 2:**
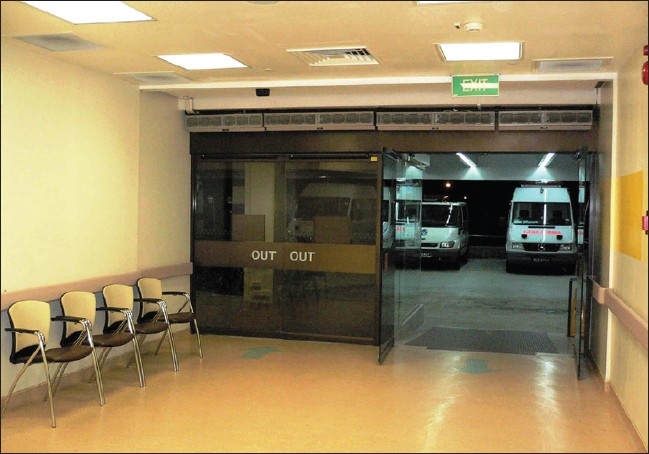
A separate new entrance to the ‘fever area’ where there is minimal air mix with the other areas of the emergency department

**Figure 3:**
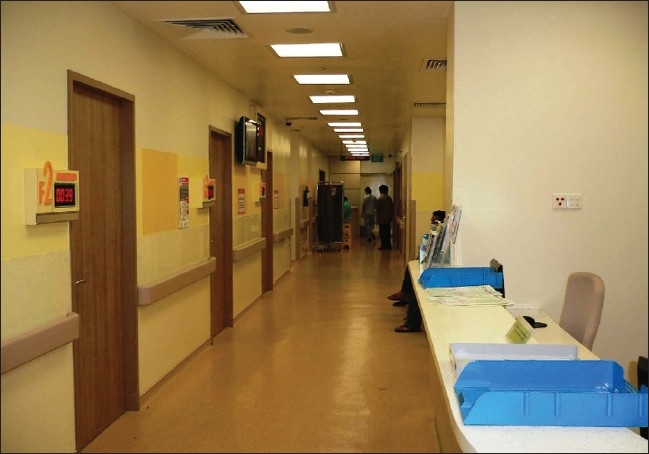
“Fever” consultation rooms with negative pressure ventilation

These areas are relatively new areas, constructed following the lessons learnt during the SARS outbreak [Figures 1 and 2]. Many healthcare systems were overwhelmed by the SARS epidemic. The system design, public health functions, equipment and supplies as well as collaborative arrangements were either not in place or not in alignment then. Existing triage areas in the ED are often designed with patient flow and satisfaction in mind, rather than healthcare workers safety and protection. As air currents may transport infections, fever and high risk patients are now being managed separately from others. The febrile areas in the ED have undergone structural re-engineering and upgrading of the ventilation system [Figure 3]. There usually exist design flaws in many hospitals, such as turbulent ventilation across patient access areas, the flow of aerosolized gases between treatment areas and the shortage or absence of negative pressure rooms. Consideration of design, equipment and ventilation are important Building ventilation, whether natural or mechanical serves to dilute droplets nuclei in the air and is the single most important engineering control in the prevention of transmission of airborne infections.[6–8] In the new fever areas, rooms with negative pressure ventilation are now available. The exhaust rate in these rooms must exceed the air supply rate by a generous margin. Infected air from patients in this area is prevented from staying in the area and circulating in the corridor, by an exhaust system that filters it to the outside environment. During the construction phase, it is essential to consult the ventilation engineer with regard to the sufficient amount of flow without causing too high turbulence.

The positive pressure gradient between the isolation cubicles/rooms and the rest of the area is approximately 15 Pa. A negative pressure room should also preferably have windows which do not open. Having ante-rooms too will help reduce the escape of droplet nuclei during opening and closing of doors. It must also be noted that patients and staff in negative pressure rooms are at increased risk in the event of a fire. This is because fire and smoke can be drawn into these rooms from the adjacent corridors or wards by reason of the differential pressures. The SARS virus is transmitted primarily by bio-aerosol droplets or direct, very close personal contact. During the outbreak when onsite measurement of bio-aerosol dispersion was done, it was able to predict the distribution fairly well, in agreement with the spatial infection pattern of SARS cases.[3]

**Figure 4:**
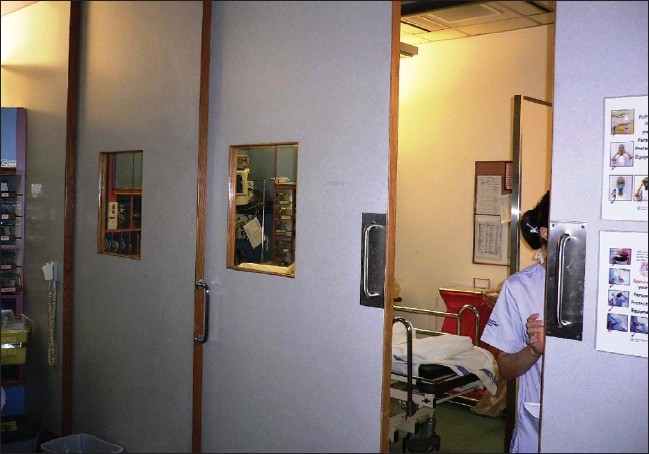
Negative pressure ventilation end cubicle in the resuscitation area with lead X-ray proof door partitions

Febrile patients who are non-ambulatory and too ill to walk are managed in the critical care/resuscitation area which has two end rooms prepared with negative pressure ventilation and separated from other cubicles with heavy lead doors [Figure 4].

The observation unit in the ED is also equipped with isolation rooms for the management of potentially high risk and infectious patients. The doors of these rooms are fitted with a self closing device. For isolation rooms with no negative pressure ventilation, it is important to have them well ventilated with adequate fresh air exchange. The hospital Infectious Diseases committee has also come up with guidelines and operating procedures on the recommendations for the admission of suitable patients to isolation rooms as well as negative pressure rooms.

**Figure 5:**
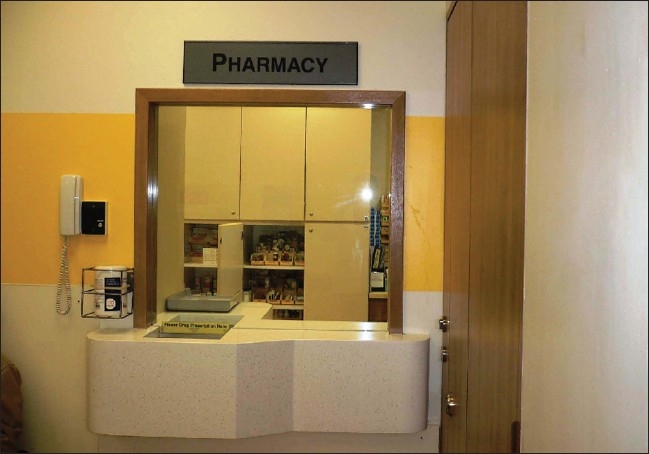
A separate emergency pharmacy in the ‘fever’ area to reduce patient movement as much as possible

**Figure 6:**
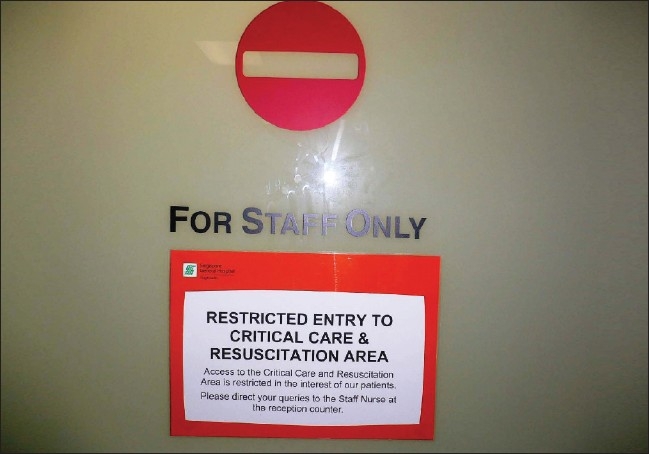
Signages and restricted entry points are important especially during outbreaks

These infrastructure changes and facilities are not going to be effective if the staff do no change their mindset and remain highly compliant with guidelines and safe practices [Figures 5 and 6]. A consolidated strategy which is multipronged is essential. These would include not just structural changes but also mechanisms for contact tracing, syndromic surveillance, proper hand washing techniques and the practice of universal precautions.

Disinfection and cleaning of the febrile areas too represent a crucial duty. Non-disposable material, equipment and work surfaces must be subjected to frequent cleaning and thus must incorporate materials with resistance to solutions and solvents as well as spread of infection. Disinfection with hypochlorite, 1000 ppm, is regularly done. This is for all wards, environment, facilities, equipment, horizontal surfaces, surfaces touched by patients and staff as well as toilet facilities. Other considerations include humidity control which can have an effect on the spread of infection such as methicillin resistant *Staphylococcus aureus* (MRSA). In fact the choice of color and ambience of the hospital areas and rooms can have an effect and psychological implications on both staff and patients treatment, management and[9–11] recovery.

## HOSPITAL DESIGN AND HAND HYGIENE

The experience of controlling SARS provided many lessons on how to prepare for a major outbreak. Improving general infection control measures and procedures as well as preparedness has the potential to enhance routine healthcare on a daily basis as well as increase our chance of a successful handling of the next pandemic.

One key component of limiting the spread of healthcare related infectious diseases is adequate infection control practice. A cornerstone of this is ensuring good hand hygiene. Hand washing has been recommended as the single most important practice to control hospital acquired infection. In isolation rooms, of observation and general wards, there are personalized hand washing facilities within each room to reduce cross-contamination. These isolation rooms help to prevent direct and even indirect contact transmission and droplet transmission. Access to examination gloves, alcohol-based hand-rub and trash containers or receptacle is also important. Many have the perception that unavailability or inadequate hand washing facilities and sinks contribute towards poor compliance. Few studies have prospectively evaluated the association between hand hygiene compliance and building plan and design. Lankford *et al*. found that hand hygiene compliance in a new healthcare facility (with more sinks provided) decreased significantly.[12] They concluded that peer and team behaviour have greater impact on good habits rather than just building design and ergonomics. This was echoed by a few other studies as well.[13,14]

## INVASIVE PROCEDURES

In performing procedures where there exists a considerable and high risk of transmission, appropriate garment and devices have to be used. These would include, N 95 masks, goggles and face shields, hair and shoe covers, impervious gowns and aprons as well as positive pressure air-powered respirator (PAPR). The latter is a hood worn over the head and face to shield the healthcare personnel from air droplets and any secretions from infected patients when performing procedures such as suctioning and endotracheal intubation.

## OTHER PRACTICES

In handling an outbreak such as SARS there must be good coordination at all levels. This would mean the communications must be excellent as well. Relevant messages, information and instructions must be disseminated effectively through an agreed upon channel or system. Notification of infectious disease cases must also be timely and this calls for staff to be highly vigilant.

At Singapore General Hospital, with the use of computerized records, it is easier to trace and track patients and information. This is important for contact tracing and syndromic surveillance. In the re-engineering of the departments, after SARS, inputs from clinicians and nurses were obtained. Infectious diseases specialists also played an important role from the inception, where they educated the engineers and architects as well as the contractors about potential infection control risks. Frequent site visits are a must. Caution must also be maintained when interpreting results from infection control literature, because the findings and recommendations are often based on retrospective investigations of infection outbreaks in particular settings and thus are tailored to those settings. They may or may not be applicable to other settings.[15,16]

## CONCLUSION

In being able to handle outbreaks well, there must be collaboration and a consolidated strategy which is understood and practiced by all. At the hospitals there must be continuity between Emergency Departments, observation wards and general wards, ICUs, isolation wards, operating theatres, laboratories and outpatient clinics. However, this must go beyond just healthcare institutions and hospitals. At the national level, it must include primary care and general practice clinics, communicable diseases centers, government services, schools, the mass media and press, immigrations department, transportation department, pharmaceutical industry, the police and many more. Frequent exercises to practice and test out our preparedness are also very crucial because then only can we learn the setbacks and correct them.

The Ministry of Health, Singapore Medical Association, College of Family Practitioners and various other healthcare organizations have created a detailed plan called the Primary Care Pandemic Framework, to help primary care clinics work with the 18 government polyclinics to provide appropriate care for influenza and non-influenza patients during a pandemic. The Framework advises on how to prepare and organize a primary care clinic for a pandemic; including modifications to clinic workflow and processes to avoid cross infection, use of personal protection equipment, hospital referral and environmental design and cleaning.[17] Both infrastructure and design have a significant effect on our work in the healthcare sectors. This issue has often been taken for granted but now is the time to make it work for us and our patients.
